# Perspectives of Health Care Professionals on Multimodal Interventions for Cancer Cachexia

**DOI:** 10.1089/pmr.2022.0045

**Published:** 2022-12-02

**Authors:** Koji Amano, Saori Koshimoto, Jane B. Hopkinson, Vickie E. Baracos, Naoharu Mori, Tatsuya Morita, Shunsuke Oyamada, Hiroto Ishiki, Eriko Satomi, Takashi Takeuchi

**Affiliations:** ^1^ Department of Palliative Medicine, National Cancer Center Hospital, Tokyo, Japan.; ^2^ Department of Palliative and Supportive Medicine, Graduate School of Medicine, Aichi Medical University, Aichi, Japan.; ^3^ School of Health Care Sciences, Faculty of Medicine, Tokyo Medical and Dental University, Tokyo, Japan.; ^4^ Department of Human Nutrition, Faculty of Human Nutrition. Tokyo Kasei Gakuin University, Tokyo, Japan.; ^5^ School of Healthcare Sciences, College of Biomedical and Life Sciences, Cardiff University, Cardiff, United Kingdom.; ^6^ Division of Palliative Care Medicine, Department of Oncology, University of Alberta, Cross Cancer Institute, Edmonton, Alberta, Canada.; ^7^ Palliative and Supportive Care Division, Seirei Mikatahara General Hospital, Hamamatsu City, Japan.; ^8^ Department of Biostatistics, JORTC Data Center, Tokyo, Japan.; ^9^ Liaison Psychiatry and Psycho-oncology Unit, Department of Psychiatry and Behavioral Sciences, Graduate School of Medical and Dental Sciences, Tokyo Medical and Dental University, Tokyo, Japan.

**Keywords:** cancer cachexia, health care professional, multimodal intervention, nationwide survey, perspective

## Abstract

**Background:**

Holistic multimodal interventions have not been established for cancer cachexia. The beliefs and perceptions of health care professionals (HCPs) based on their experiences influence the interventions.

**Objectives:**

HCPs' knowledge, perceptions, and practices in cancer cachexia management were evaluated.

**Design/Setting/Subjects/Measurements:**

A nationwide questionnaire survey was conducted that focused on the perspectives of HCPs on interventions in 451 designated cancer hospitals across Japan. Descriptive statistics were applied.

**Results:**

Among 2255 participants, 1320 responded (58.5%), and 1188 in 258 institutes were included in the analysis. The current international definition of cancer cachexia is not commonly known and recent clinical practice guidelines have not been widely adopted. More than 50% of participants considered ≥5% weight loss in six months and ECOG PS (Eastern Cooperative Oncology Group Performance Status) 2–4 to be cancer cachexia, whereas 50% answered that there was no relationship between life expectancy and cancer cachexia. Participants tended to consider it important to initiate nutritional and exercise interventions before cancer cachexia becomes apparent. The majority of participants recognized the importance of holistic multimodal interventions, particularly for the management of physical and psychological symptoms; however, only 20% reported that they educated patients and families. Furthermore, 33% of participants considered themselves to have provided patients and families with sufficient nutritional and exercise interventions and evidence-based information.

**Conclusion:**

The results reveal that HCPs are not regularly providing education and emotional support to patients and families suffering from cancer cachexia. The results also show the need for education for HCPs to enhance implementation of holistic multimodal interventions for cancer cachexia.

## Introduction

Patients with cancer cachexia develop a multifactorial syndrome that involves the ongoing loss of body weight and skeletal muscle mass with progressive functional impairments that cannot be fully reversed by usual nutritional support.^1,2^ Cancer cachexia is characterized by negative protein and energy balances owing to the combination of a reduced dietary intake and abnormal metabolism.^1,2^

Accumulating evidence has shown that systemic inflammation is involved in the mechanisms responsible for cancer cachexia and that proinflammatory cytokines generate various cachexia-related physical and psychological symptoms through alternations in the central nervous system in patients with advanced cancer.^3,4^ In addition, these symptoms are worsened by cancer treatments and are associated with the emotional distress experienced by patients and families.^4–6^ Therefore, the latest evidence-based clinical practice guidelines on the management of cancer cachexia, clinical nutrition in cancer, and end-of-life care for cancer patients have advocated holistic multimodal interventions, which is an approach addressing not only physical health but also psychological, emotional, and social well-being issues by all health care professionals (HCPs) including psychologists and social workers, to meet the physiological and psychological needs of patients and families despite the lack of a clear mandate for diagnosis and treatment of the cachexia-related problems.^7–10^ Although such multimodal approach attends to restoring or sustaining the physical body, it also alleviates distress and supports connectedness with others.^4–6^

However, limited information is currently available on the effectiveness of holistic multimodal interventions for cancer cachexia,^7–10^ and these interventions have not been established although an ideal multimodal care team has already been conceptualized.^8^ Moreover, the findings of three global surveys among HCPs involved in cancer cachexia management revealed that they desired more specific interventions to increase the quality of life of patients.^11^ The findings of surveys also underscored the need for increased awareness of cancer cachexia and its management among HCPs.^11^ Furthermore, the international survey, as a part of the Global Educational Needs Evaluation, a systemic interprofessional study in cancer cachexia (GENESIS-CC), was recently conducted by the Society on Sarcopenia, Cachexia and Wasting Disorders to establish an international cancer cachexia education program.^12^

Based on these findings, we recently suggested a specific approach that addresses not only physical health, but also psychological, emotional, and social well-being issues among patients with advanced cancer and families.^4^ However, several issues have yet to be resolved, such as the interventions that need to be prioritized and the roles that need to be adopted by members of the multimodal care team. Therefore, we conducted a nationwide questionnaire survey in Japan that focused on the perspectives of HCPs on holistic multimodal interventions for cancer cachexia. The survey was based on our suggestion^4^ that insights into the lived experiences of HCPs supporting patients with cancer and families can help to tailor holistic multimodal interventions to mitigate the impact of cancer cachexia and attenuate the emotional distress of patients and families. We also assessed the knowledge of HCPs on cancer cachexia, status considered as cancer cachexia by HCPs, and daily clinical practices performed by HCPs.

## Methods

### Sites and participants

A nationwide multicenter self-report questionnaire survey was conducted in 451 designated cancer hospitals across Japan between February and March 2022. Designated cancer hospitals are a nationwide network of medical centers appointed by the Ministry of Health, Labour and Welfare in Japan. We sent a document explaining the objective of this study and five questionnaires to the director of each institute. We then asked him/her to invite five HCPs (a physician, pharmacist, nurse, dietician, and either a physical therapist, occupational therapist, speech therapist, psychologist, or social worker) who are responsible for patients with cancer and families to respond to the questionnaires within one week, because we did not have any staff information of each institute. We also sent a reminder after two weeks, and the survey was closed two weeks thereafter.

Inclusion criteria were as follows: all participants had to (1) have at least three years of practicing experience and (2) care for patients with cancer as their primary specialty.

### Ethics

Potential participants were informed that the survey used anonymized questionnaires and the results obtained were to be analyzed with confidentiality in the invitation letter. The completion and return of the questionnaire were regarded as consent to participate in this study. If participants did not want to participate, we requested the return of the questionnaire with “no participation” indicated. If participants were not eligible to participate in this survey, we also requested the return of the questionnaire with “not eligible” indicated.

Approval from the Institutional Review Board of the National Cancer Center was not required according to national policies in Japan because this study (Institute Research Number: 6000-050) was a minimal risk study involving only HCPs and was beyond the scope of the Ethical Guidelines for Medical and Health Research Involving Human Subjects in Japan.

### Questionnaire

The anonymized self-report questionnaire for HCPs in Japanese for this study was developed by the authors. Items included in the questionnaire were based on a previous survey^11^ and discussions among the authors. The face validity of the questionnaire was confirmed by a pilot test with two physicians, two nurses, one dietician, and one psychologist in the National Cancer Center Hospital.

### Participant demographics

Data were obtained on participant demographics and clinical experience, for example, age, sex, occupation, practicing experience, primary area of practice, and participating institute information, including hospital location and number of beds.

### Knowledge of cancer cachexia

We asked HCPs about their knowledge on the current international definition of cancer cachexia^1^ and clinical practice guidelines on the management of cancer cachexia^7–9^ with yes or no questions. We also asked if HCPs used the definition and guidelines in their daily clinical practices with yes or no questions.

### Status of cancer cachexia and nutritional and exercise interventions

We asked HCPs about the status considered as cancer cachexia regarding weight loss in six months, the Eastern Cooperative Oncology Group Performance Status (ECOG PS), and life expectancy because these factors are considered to be important in the current classification of cancer cachexia.^1^ We also asked HCPs to select one of the several factors in each item regarding weight loss in six months, ECOG PS, and life expectancy that indicated the need for the initiation of nutritional and exercise interventions.

### Holistic multimodal interventions

We asked HCPs to evaluate nine components of holistic multimodal interventions for cancer cachexia based on our suggestions^4^ using the following seven-point Likert scale: (1) absolutely disagree, (2) disagree, (3) somewhat disagree, (4) neither agree nor disagree, (5) somewhat agree, (6) agree, and (7) absolutely agree. The first question was “how important is each of the 9 items in cancer cachexia management?” and the second was “how much do you perform each of the 9 items in daily clinical practices?”

### Beliefs and perceptions on cancer cachexia

We asked 10 questions about beliefs and perceptions on cancer cachexia management by HCPs using the following seven-point Likert scale: (1) absolutely disagree, (2) disagree, (3) somewhat disagree, (4) neither agree nor disagree, (5) somewhat agree, (6) agree, and (7) absolutely agree.

### Statistical analysis

Participant demographics and clinical experience were presented as numbers (%) for categorical variables or median (interquartile range) for continuous variables where appropriate. Scores were presented as mean ± standard deviation. Comparisons among the groups were performed using the Mantel–Haenszel test for trends for numbers (%) or the Kruskal–Wallis test for medians where appropriate. All results were considered to be significant if the *p*-value was <0.05. All analyses were performed using SPSS software version 27.0.

## Results

A total of 2255 participants at 451 institutes were asked to participate in this survey, and 1320 responded (response rate, 58.5%). Among these, the number of questionnaires with “no participation” was 52, whereas that with “not eligible” was 72. Eight participants were excluded owing to missing data. Therefore, 1188 participants in 258 institutes were included in the analysis.

### Participant demographics

Data on participant demographics and clinical experience and participating institute information are summarized in Tables 1 and 2.

**Table 1.** Participant Demographics and Clinical Experience

	Total (***N*** = 1188)	Physician (***n*** = 236)	Pharmacist (***n*** = 246)	Nurse (***n*** = 247)	Dietician (***n*** = 237)	PT/OT/ST (***n*** = 122)	Psychologist (***n*** = 36)	Social worker (***n*** = 64)	***p***
Age, years	44.0 (38.0–51.0)	54.0 (48.0–60.0)	39.0 (34.0–44.0)	46.0 (42.0–51.0)	42.0 (36.0–49.5)	43.0 (37.0–48.3)	39.0 (36.0–44.0)	42.0 (37.0–49.0)	<0.001
Sex									
Male	459 (38.6)	192 (81.4)	139 (56.5)	11 (4.5)	21 (8.9)	68 (55.7)	13 (36.1)	15 (23.4)	<0.001
Female	710 (59.8)	36 (15.3)	103 (41.9)	234 (94.7)	214 (90.3)	53 (43.4)	22 (61.1)	48 (75.0)	
Practicing experience									
3–4 years	30 (2.5)	0 (0.0)	11 (4.5)	0 (0.0)	10 (4.2)	2 (0.0)	3 (8.3)	4 (6.3)	<0.001
5–9 years	154 (13.0)	11 (4.7)	61 (24.8)	4 (1.6)	38 (16.0)	10 (8.2)	11 (30.6)	19 (29.7)	
10–19 years	415 (34.9)	42 (17.8)	108 (43.9)	77 (31.2)	90 (38.0)	50 (41.0)	18 (50.0)	30 (46.9)	
20 years or more	582 (49.0)	182 (77.1)	64 (26.0)	165 (66.8)	98 (41.4)	59 (48.4)	3 (8.3)	11 (17.2)	
Practicing experience of cancer care									
1–2 years	68 (5.7)	1 (0.4)	18 (7.3)	1 (0.4)	24 (10.1)	14 (11.5)	6 (16.7)	4 (6.3)	<0.001
3–4 years	81 (6.8)	6 (2.5)	26 (10.6)	0 (0.0)	24 (10.1)	11 (9.0)	3 (8.3)	11 (17.2)	
5–9 years	285 (24.0)	22 (9.3)	78 (31.7)	19 (7.7)	85 (35.9)	43 (35.2)	16 (44.4)	22 (34.4)	
10–19 years	481 (40.5)	83 (35.2)	109 (44.3)	131 (53.0)	82 (34.6)	45 (36.9)	10 (27.8)	21 (32.8)	
20 years or more	264 (22.2)	123 (52.1)	14 (5.7)	96 (38.9)	18 (7.6)	7 (5.7)	0 (0.0)	6 (9.4)	
No. of patients with advanced cancer/month									
1–9	212 (17.8)	15 (6.4)	36 (14.6)	18 (7.3)	68 (28.7)	43 (35.2)	13 (36.1)	19 (29.7)	<0.001
10–19	379 (31.9)	64 (27.1)	76 (30.9)	70 (28.3)	89 (37.6)	47 (38.5)	11 (30.6)	22 (34.4)	
20–49	398 (33.5)	100 (42.4)	90 (36.6)	95 (38.5)	60 (25.3)	26 (21.3)	10 (27.8)	17 (26.6)	
50–99	125 (10.5)	38 (16.1)	28 (11.4)	38 (15.4)	14 (5.9)	3 (2.5)	0 (0.0)	4 (6.3)	
100 or more	56 (4.7)	16 (6.8)	10 (4.1)	25 (10.1)	5 (2.1)	0 (0.0)	0 (0.0)	0 (0.0)	
Primary area of practice									
Palliative care	551 (46.4)	128 (54.2)	96 (39.0)	198 (80.2)	67 (28.3)	19 (15.6)	22 (61.1)	21 (32.8)	<0.001
Cancer treatment	406 (34.2)	101 (42.8)	126 (51.2)	38 (15.4)	82 (34.6)	54 (44.3)	1 (2.8)	4 (6.3)	
Other	213 (17.9)	6 (2.5)	20 (8.1)	10 (4.0)	82 (34.6)	47 (38.5)	11 (30.6)	37 (57.8)	

Values are given *n* (%) or median (interquartile range). Comparisons among the groups were performed using the Mantel–Haenszel test for trends for *n* (%) or the Kruskal–Wallis test for medians where appropriate.

OT, occupational therapist; PT, physical therapist; ST, speech therapist.

**Table 2.** Participating Institute Information (*n* = 258)

Hospital location	
Metropolitan city	18 (7.0)
Ordinance-designated city	62 (24.0)
Core city	81 (31.4)
Other	97 (37.6)
No. of hospital beds	
200 or less	6 (2.3)
200–300	26 (10.1)
300–500	84 (32.6)
500 or more	142 (55.0)
Palliative care team, yes	255 (98.8)
Palliative care unit, yes	76 (29.5)

Values are given as *n* (%).

The majority of participants were men among physicians (81.4%) and women among nurses and dieticians (94.7% and 90.3%, respectively). The majority of participants were experienced HCP. The number of patients with advanced cancer treated per month was lower among dieticians, physical therapists/occupational therapists/speech therapists, psychologists, and social workers than among physicians, pharmacists, and nurses. Regarding the primary area of practice, the majority of physicians, pharmacists, and nurses were involved in palliative care or cancer treatment (97.0%, 90.2%, and 95.6%, respectively), whereas 30.6–57.8% of dieticians, physical therapists/occupational therapists/speech therapists, psychologists, and social workers were engaged in other areas (Table 1).

Designated cancer hospitals were located in big cities across Japan, with more than half of the participating institutes having 500 or more beds and one third having 300–500 beds. The majority of participating institutes had a palliative care team (98.8%), whereas less than one third had a palliative care unit (Table 2).

### Knowledge of cancer cachexia

The results obtained on the knowledge of cachexia in HCPs and their utilization of the definition and clinical practice guidelines are given in Table 3.

**Table 3.** Knowledge and Utilization of the Definition and Clinical Practice Guidelines

	Total (***N*** = 1188)	Physician (***n*** = 236)	Pharmacist (***n*** = 246)	Nurse (***n*** = 247)	Dietician (***n*** = 237)	PT/OT/ST (***n*** = 122)	Psychologist (***n*** = 36)	Social worker (***n*** = 64)	***p***
Fearon et al^1^									
Knowledge, yes	568 (47.8)	127 (53.8)	112 (45.5)	119 (48.2)	138 (58.2)	58 (47.5)	5 (13.9)	9 (14.1)	<0.001
Utilization, yes	322 (27.1)	66 (28.0)	67 (27.2)	54 (21.9)	104 (43.9)	27 (22.1)	3 (8.3)	1 (1.6)	0.020
Roeland et al^7^									
Knowledge, yes	285 (24.0)	83 (35.2)	76 (30.9)	70 (28.3)	31 (13.1)	24 (19.7)	0 (0.0)	1 (1.6)	<0.001
Utilization, yes	89 (7.5)	30 (12.7)	23 (9.3)	11 (4.5)	13 (5.5)	12 (9.8)	0 (0.0)	0 (0.0)	0.002
Arends et al^8^									
Knowledge, yes	155 (13.0)	62 (26.3)	37 (15.0)	26 (10.5)	19 (8.0)	11 (9.0)	0 (0.0)	0 (0.0)	<0.001
Utilization, yes	44 (3.7)	20 (8.5)	10 (4.1)	3 (1.2)	8 (3.4)	3 (2.5)	0 (0.0)	0 (0.0)	<0.001
Muscaritoli et al^9^									
Knowledge, yes	275 (23.1)	62 (26.3)	44 (17.9)	42 (17.0)	111 (46.8)	16 (13.1)	0 (0.0)	0 (0.0)	0.020
Utilization, yes	137 (11.5)	24 (10.2)	18 (7.3)	15 (6.1)	73 (30.8)	7 (5.7)	0 (0.0)	0 (0.0)	0.881

Values are given *n* (%). Comparisons among the groups were performed using the Mantel–Haenszel test for trends.

The current international definition of cancer cachexia,^1^ which was published in 2011, is not commonly known in Japan. Only 47.8% knew the definition in all subjects, and the number of psychologists and social workers aware of the definition was markedly smaller than that of the other occupations (13.9% and 14.1%, respectively). Moreover, implementation of the recent clinical practice guidelines^7–9^ has not yet become widespread (7.5%, 3.7%, and 11.5%, respectively).

### Status of cancer cachexia and nutritional and exercise interventions

The status considered as cancer cachexia and the initiation of nutritional and exercise interventions are described in Table 4.

**Table 4.** Status Considered as Cancer Cachexia and Initiation of Nutritional and Exercise Interventions (*N* = 1188)

	Status considered as cancer cachexia	Initiation of nutritional and exercise interventions
Weight loss in 6 months		
<2%	2 (0.2)	58 (4.9)
≥2%	21 (1.8)	238 (20.0)
≥5%	764 (64.3)	648 (54.5)
≥10%	320 (26.9)	209 (17.6)
≥15%	35 (2.9)	11 (0.9)
≥20%	33 (2.8)	9 (0.8)
Eastern Cooperative Oncology Group Performance Status		
0–4	18 (1.5)	113 (9.5)
1–4	158 (13.3)	478 (40.2)
2–4	629 (52.9)	498 (41.9)
3–4	350 (29.5)	79 (6.6)
4	8 (0.7)	1 (0.1)
Life expectancy		
<1 week	2 (0.2)	2 (0.2)
<2 weeks	9 (0.8)	2 (0.2)
<1 month	93 (7.8)	18 (1.5)
<3 months	307 (25.8)	84 (7.1)
<6 months	163 (13.7)	127 (10.7)
No relationship	598 (50.3)	945 (79.5)

Values are given as *n* (%).

About 64.3% of participants considered ≥5% weight loss in six months to be significant in cancer cachexia, and 54.5% considered ≥5% weight loss in six months to be significant in the initiation of nutritional and exercise interventions. Furthermore, 26.9% of participants regarded ≥10% weight loss as cancer cachexia, whereas 20.0% considered it important to initiate nutritional and exercise interventions with ≥2% weight loss.

About 52.9% of participants considered ECOG PS 2–4 as cancer cachexia, whereas 40.2% and 41.9% indicated the necessity of initiating nutritional and exercise interventions at ECOG PS 1 and 2, respectively.

Half of the participants answered that there was no relationship between life expectancy and cancer cachexia, whereas 25.8% considered cancer cachexia to be associated with a less than three months life expectancy. Furthermore, 79.5% answered that there was no relationship between life expectancy and the initiation of nutritional and exercise interventions.

Participants were likely to consider that cancer cachexia was associated with decreasing weight and worsening ECOG PS. They also seemed to consider it important to initiate nutritional and exercise interventions before cancer cachexia becomes apparent regardless of life expectancy.

### Holistic multimodal interventions

**FIG. 1.**
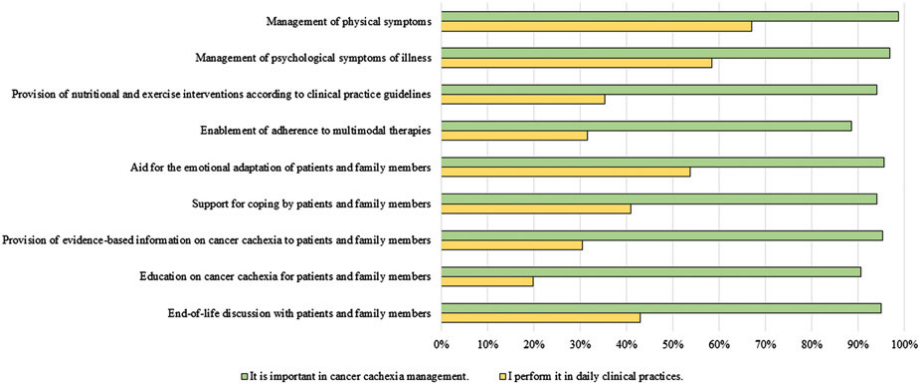
Evaluation of holistic multimodal interventions for cancer cachexia in all occupations (*N* = 1188). The percentages that represent the number of health care professionals giving a rating of 5 (somewhat agree), 6 (agree), and 7 (absolutely agree) in each of 9 items are shown.

Regarding evaluations of holistic multimodal interventions for cancer cachexia in all occupations, the percentages that represent the number of HCPs giving a rating of 5 (somewhat agree), 6 (agree), and 7 (absolutely agree) in each of 9 components are given in Figure 1. In addition, the results of comparisons of mean scores among 7 occupations are given in Table 5.

**Table 5.** Comparison of Perspectives on Holistic Multimodal Interventions for Cancer Cachexia Among Occupations

	Total (***N*** = 1188)	Physician (***n*** = 236)	Pharmacist (***n*** = 246)	Nurse (***n*** = 247)	Dietician (***n*** = 237)	PT/OT/ST (***n*** = 122)	Psychologist (***n*** = 36)	Social worker (***n*** = 64)	***p***
It is important in cancer cachexia management.									
Management of physical symptoms	**6.5 ± 0.6**	**6.4 ± 0.7**	**6.4 ± 0.7**	**6.7 ± 0.5**	**6.6 ± 0.5**	**6.6 ± 0.5**	**6.7 ± 0.5**	**6.4 ± 0.7**	<0.001
Management of psychological symptoms of illness	**6.4 ± 0.7**	**6.2 ± 0.8**	**6.1 ± 0.9**	**6.5 ± 0.7**	**6.6 ± 0.6**	**6.5 ± 0.6**	**6.4 ± 0.7**	**6.4 ± 0.7**	<0.001
Provision of nutritional and exercise interventions according to clinical practice guidelines	**6.0 ± 0.8**	**5.8 ± 0.9**	**6.1 ± 0.7**	**6.0 ± 0.8**	**5.9 ± 0.9**	**6.2 ± 0.8**	**6.4 ± 0.7**	**6.0 ± 0.9**	<0.001
Enablement of adherence to multimodal therapies	**5.7 ± 0.9**	**5.5 ± 1.1**	**5.7 ± 0.9**	**5.7 ± 0.9**	**5.8 ± 0.9**	**5.9 ± 0.9**	**5.8 ± 1.1**	**5.9 ± 0.9**	0.016
Aid for the emotional adaptation of patients and family members	**6.1 ± 0.8**	**6.0 ± 0.9**	**5.8 ± 0.9**	**6.3 ± 0.7**	**6.4 ± 0.7**	**6.2 ± 0.8**	**5.8 ± 1.0**	**6.3 ± 0.7**	<0.001
Support for coping by patients and family members	**6.0 ± 0.8**	**5.9 ± 0.9**	**5.7 ± 0.9**	**6.2 ± 0.7**	**6.2 ± 0.7**	**6.1 ± 0.9**	**5.7 ± 1.0**	**6.3 ± 0.8**	<0.001
Provision of evidence-based information on cancer cachexia to patients and family members	**6.1 ± 0.8**	**5.9 ± 0.9**	**6.0 ± 0.8**	**6.2 ± 0.8**	**6.1 ± 0.8**	**6.2 ± 0.7**	**6.4 ± 0.7**	**6.3 ± 0.7**	<0.001
Education on cancer cachexia for patients and family members	**5.9 ± 0.9**	**5.7 ± 1.0**	**5.9 ± 0.9**	**6.0 ± 1.0**	**5.8 ± 0.9**	**6.0 ± 0.8**	**6.2 ± 0.9**	**6.0 ± 0.8**	0.005
End-of-life discussions with patients and family members	**6.3 ± 0.8**	**6.2 ± 0.9**	**6.2 ± 0.9**	**6.4 ± 0.8**	**6.4 ± 0.8**	**6.3 ± 0.8**	**6.1 ± 1.1**	**6.4 ± 0.8**	0.005
I perform it in daily clinical practices.									
Management of physical symptoms	4.7 ± 1.4	**5.5 ± 0.8**	4.8 ± 1.1	**5.4 ± 0.8**	3.5 ± 1.3	**5.1 ± 0.7**	3.0 ± 1.3	2.9 ± 1.4	<0.001
Management of psychological symptoms of illness	4.5 ± 1.2	4.9 ± 1.0	4.0 ± 1.2	**5.2 ± 0.8**	3.8 ± 1.3	4.7 ± 0.9	**5.1 ± 0.9**	4.0 ± 1.4	<0.001
Provision of nutritional and exercise interventions according to clinical practice guidelines	3.8 ± 1.4	3.9 ± 1.3	3.1 ± 1.4	3.7 ± 1.2	4.6 ± 1.2	4.5 ± 1.2	2.1 ± 1.3	2.7 ± 1.5	<0.001
Enablement of adherence to multimodal therapies	3.9 ± 1.2	4.3 ± 1.1	4.0 ± 1.2	4.1 ± 1.0	3.5 ± 1.3	3.8 ± 1.1	3.1 ± 1.4	3.1 ± 1.4	<0.001
Aid for the emotional adaptation of patients and family members	4.3 ± 1.3	4.7 ± 1.1	3.4 ± 1.3	**5.1 ± 0.8**	3.8 ± 1.4	4.3 ± 0.9	**5.4 ± 0.7**	4.5 ± 1.3	<0.001
Support for coping by patients and family members	4.0 ± 1.3	4.5 ± 1.2	3.2 ± 1.3	4.8 ± 0.9	3.5 ± 1.3	3.9 ± 1.1	**5.1 ± 1.1**	4.0 ± 1.4	<0.001
Provision of evidence-based information on cancer cachexia to patients and family members	3.6 ± 1.4	4.2 ± 1.3	3.2 ± 1.4	3.9 ± 1.3	3.6 ± 1.4	3.4 ± 1.3	2.2 ± 1.2	2.9 ± 1.3	<0.001
Education on cancer cachexia for patients and family members	3.2 ± 1.4	3.9 ± 1.4	2.9 ± 1.3	3.5 ± 1.3	3.2 ± 1.4	3.1 ± 1.3	2.0 ± 1.1	2.3 ± 1.2	<0.001
End-of-life discussions with patients and family members	3.9 ± 1.7	**5.0 ± 1.2**	2.8 ± 1.4	**5.1 ± 1.1**	2.6 ± 1.3	3.3 ± 1.4	4.8 ± 1.2	4.9 ± 1.2	<0.001

Values are given as mean ± standard deviation. Comparisons among the groups were performed using the Kruskal–Wallis test. Questionnaire items were rated on a scale 1–7, with 1 indicating “absolutely disagree” and 7 indicating “absolutely agree.” Values in bold are 5 indicating “somewhat agree” or more.

The majority of participants recognized the importance of all items, particularly the management of the physical and psychological symptoms of illness. However, only 19.9% considered themselves to have educated patients and families on cancer cachexia. Furthermore, only 33.3% of participants considered themselves to have provided patients and families with sufficient nutritional and exercise interventions and evidence-based information on cancer cachexia (Fig. 1).

Physicians, nurses, and physical therapists/occupational therapists/speech therapists were involved in the management of physical symptoms, whereas nurses and psychologists were engaged in the management of the psychological symptoms of illness and provision of aid for the emotional adaptation of patients and families. Psychologists contributed to support for coping by patients and families. Physicians and nurses actively worked in end-of-life discussions with patients and families (Table 5).

### Beliefs and perceptions on cancer cachexia

The results of the evaluation of beliefs and perceptions on cancer cachexia management by HCPs and comparisons among the seven occupations are given in Table 6.

**Table 6.** Comparison of Beliefs and Perceptions on Cancer Cachexia Management Among Occupations

	Total (***N*** = 1188)	Physician (***n*** = 236)	Pharmacist (***n*** = 246)	Nurse (***n*** = 247)	Dietician (***n*** = 237)	PT/OT/ST (***n*** = 122)	Psychologist (***n*** = 36)	Social worker (***n*** = 64)	***p***
I have knowledge on cancer cachexia management	4.0 ± 1.4	4.6 ± 1.2	3.7 ± 1.3	4.3 ± 1.2	4.2 ± 1.3	3.9 ± 1.2	2.2 ± 1.2	2.7 ± 1.3	<0.001
I have experience of cancer cachexia management	4.2 ± 1.5	4.8 ± 1.2	3.6 ± 1.5	4.7 ± 1.3	4.3 ± 1.5	4.2 ± 1.4	2.3 ± 1.4	2.6 ± 1.4	<0.001
I have confidence in cancer cachexia management	3.0 ± 1.3	3.6 ± 1.3	2.5 ± 1.2	3.2 ± 1.3	3.1 ± 1.3	3.0 ± 1.2	1.7 ± 0.9	2.0 ± 1.0	<0.001
My role is important in cancer cachexia management	4.8 ± 1.3	**5.0 ± 1.2**	4.3 ± 1.2	**5.1 ± 1.1**	**5.2 ± 1.0**	4.8 ± 1.1	3.7 ± 1.2	3.5 ± 1.4	<0.001
I play a role in cancer cachexia management	3.6 ± 1.3	4.2 ± 1.2	3.0 ± 1.3	3.6 ± 1.3	4.0 ± 1.3	3.7 ± 1.2	2.5 ± 1.3	2.8 ± 1.3	<0.001
Sharing information on the patient in the multimodal care team is essential	**6.0 ± 0.9**	**5.9 ± 1.0**	**6.0 ± 0.9**	**6.1 ± 0.8**	**6.3 ± 0.9**	**6.0 ± 0.9**	**5.8 ± 0.8**	**5.6 ± 1.3**	<0.001
We share information on the patient in the multimodal care team	4.5 ± 1.5	4.7 ± 1.3	4.0 ± 1.5	4.6 ± 1.4	**5.1 ± 1.4**	4.5 ± 1.3	3.0 ± 1.6	3.9 ± 1.6	<0.001
Training for cancer cachexia management is vital for health care professionals in cancer care	**5.7 ± 1.0**	**5.5 ± 1.0**	**5.9 ± 0.9**	**5.7 ± 1.0**	**6.0 ± 0.9**	**5.7 ± 0.9**	**5.4 ± 1.0**	**5.1 ± 1.2**	<0.001
I have received training for cancer cachexia management	2.7 ± 1.6	3.0 ± 1.6	2.2 ± 1.3	2.9 ± 1.7	3.1 ± 1.7	2.8 ± 1.6	1.5 ± 1.0	2.0 ± 1.3	<0.001
My hospital provides health care professionals with training for cancer cachexia management	2.9 ± 1.5	2.9 ± 1.5	2.6 ± 1.3	2.5 ± 1.4	3.5 ± 1.6	2.8 ± 1.3	2.7 ± 1.3	2.9 ± 1.4	<0.001

Values are given as mean ± standard deviation. Comparisons among the groups were performed using the Kruskal–Wallis test. Questionnaire items were rated on a scale 1–7, with 1 indicating “absolutely disagree” and 7 indicating “absolutely agree.” Values in bold are 5 indicating “somewhat agree” or more.

Physicians, nurses, and dieticians considered their roles to be important in cancer cachexia management, whereas psychologists and social workers did not. All occupations agreed that sharing information on the patient in the multidisciplinary care team was essential and that training for cancer cachexia management was vital for HCPs in cancer care. None of the occupations considered themselves to have received adequate training, and they did not have confidence in cancer cachexia management (Table 6).

## Discussion

This is the first survey of a diverse group of Japanese HCPs who care for cancer patients and families to investigate their perspectives on holistic multimodal interventions for cancer cachexia. The results obtained revealed that the comprehensive clinical experiences of 1188 HCPs demonstrated significant gaps in knowledge of cancer cachexia as well as implementation of guideline recommendations to address it. Owing to the limited empirical research among HCPs, their beliefs and perceptions will contribute to the body of knowledge and development of holistic multimodal interventions for cancer cachexia.

To date, we have an informal consensus based only on low-quality evidence that supports an argument that nutritional interventions for patients with cancer cachexia can improve their energy and protein intake along with their emotional well-being.^7–10^ Furthermore, there has been limited evidence to support pharmacological interventions and other interventions for the management of cancer cachexia.^7–10^ However, cancer cachexia is potentially preventable and cachexia-related problems and symptoms can be managed by patient-tailored nutritional support when addressed in a timely manner,^13,14^ and patients with cancer cachexia may benefit from exercise programs to increase skeletal muscle mass and improve physical function.^15,16^

The move to study patients at an earlier stage of the cancer cachexia pathway has been driven by the argument that multimodal interventions may work for patients with precachexia and cachexia but not refractory cachexia. At present, studies to evaluate the impact of a multimodal intervention on patients with cancer cachexia using nutrition, nonsteroidal anti-inflammatory drugs/anamorelin, and exercise are underway.^17–20^ Patients targeted by these multimodal interventions are those in the precachexia and cachexia phases, whereas patients in the refractory cachexia phase and families may not be included. Therefore, holistic multimodal interventions for not only patients with advanced cancer cachexia, but also families need to be concurrently established.^4^

The results of this survey indicate that holistic multimodal interventions need to address psychological, emotional, and social well-being issues as well as physical health issues, and these interventions also need to be comprehensively and cooperatively performed by the multimodal care team. Physicians and nurses have to contribute to the interventions as not only specialists but also generalists, whereas other occupations need to play specific roles as specialists with a high level of expertise. In addition, nurses are the major health care workforce in terms of numbers, and they routinely assess items specific to cancer cachexia with or without awareness.^21^ Therefore, nurses have an important role in the management of cancer cachexia. There is potential to develop nurse-led multimodal interventions, such as the Macmillan Approach to Weight and Eating^22^ and the PiCNIC,^23,24^ and for nurses to coordinate hubs of multimodal care teams.^25^

Furthermore, patients and families also need to understand the importance of holistic multimodal interventions for cancer cachexia to achieve higher motivation for self-care and increased adherence. It is important that patients, families, and HCPs together become actively involved in the decision-making process and/or end-of-life discussion to mitigate cachexia-related distress. However, there may be not enough HCPs available to provide all the palliative care services required by patients and families. This workforce shortage needs to be addressed by increasing funding for HCP training programs in palliative care. Furthermore, there may be variability in the scope of services and degree of engagement in cancer cachexia management by HCPs. Support is needed not only for education in cancer cachexia but also to develop a more robust infrastructure of cancer cachexia clinics.

Although this was a large-scale nationwide survey, there are several limitations. Because survey participants were limited to HCPs belonging to designated cancer hospitals, the results obtained cannot be generalized. However, the management of cancer cachexia needs to be concurrently initiated from cancer care in designated cancer hospitals. Although the surveys were administered to workers at designated cancer hospitals, the number of cancer patients treated was lower among dieticians, physical therapists/occupational therapists/speech therapists, psychologists, and social workers. This may be because they were also engaged in caring for patients with other diseases, including diabetes and cerebral infarction.

Moreover, although there are guideline recommendations that recommend addressing cancer cachexia with holistic multimodal interventions, these recommendations are not yet based on solid clinical evidence.^7–10^ Furthermore, there are currently no available validated tools to evaluate holistic multimodal interventions for cancer cachexia; therefore, the present results may have been affected by acquiescence bias or agreement bias, which is common to survey research in which respondents tend to choose positive response options more frequently. However, a combination of single response items and rating items may reduce the risk of acquiescence bias.

Further studies are needed (1) to provide HCPs who are responsible for patients with cancer and families with specific information on cancer cachexia, (2) to provide HCPs with knowledge on physical and psychological symptoms and psychosocial distress experienced by patients and families, (3) to encourage HCPs to identify and target interventions for the issues experienced by patients and families for supportive and palliative care, which is comprehensively and cooperatively performed by the multimodal care team, and (4) to encourage HCPs to establish holistic multimodal interventions because there is limited evidence to support such interventions.

## Conclusion

HCPs are not regularly providing education and emotional support to patients and families suffering from cancer cachexia. The results also show the need for education for HCPs to enhance implementation of holistic multimodal interventions and the need for further development of the interventions.

## Data Availability

The datasets generated and/or analyzed in this study were not publicly available.

## Abbreviations Used

ECOG PS: Eastern Cooperative Oncology Group Performance Status
GENESIS-CC: Global Educational Needs Evaluation: a systemic interprofessional study in cancer cachexia
HCPs: health care professionals
